# Supplementary material for: Protocol for an independent patient data meta-analysis of prophylactic mesh placement for incisional hernia prevention after abdominal aortic aneurysm surgery: a collaborative European Hernia Society project (I-PREVENT-AAA)
Source: BMJ Open. 2024 Apr 16;14(4):e081046. doi: 10.1136/bmjopen-2023-081046 (PMC11029178; doi:10.1136/bmjopen-2023-081046)
Supplement: Supplementary data [file bmjopen-2023-081046supp002.pdf]

Suppl. A: Search strategies of the used databases

medline ALL Ovid

(Aortic Aneurysm, Abdominal / OR Aortic Aneurysm / OR ((Aneurysm /) AND Aorta, Abdominal /) OR ((aort* ADJ3 aneurysm*) OR aaa).ab,ti.) AND (Surgical Mesh / OR (mesh* OR dynamesh* OR vitamesh* OR surgimesh*).ab,ti.) AND (exp Preventive Health Services / OR prevention.fx. OR (prevent* OR prophyla* OR augment* OR reinforce*).ab,ti.)

embase.com

('abdominal aortic aneurysm'/exp OR 'aortic aneurysm'/de OR ((aneurysm/de OR 'aneurysm surgery'/de) AND 'abdominal aorta'/de) OR ((aort* NEAR/3 aneurysm*) OR aaa):Ab,ti) AND ('surgical mesh'/exp OR (mesh* OR dynamesh* OR vitamesh* OR surgimesh*):ab,ti) AND (prophylaxis/de OR prevention/de OR prevention:lnk OR (prevent* OR prophyla* OR augment* OR reinforce*):ab,ti)

Web of science

TS=((((aort* NEAR/2 aneurysm*) OR aaa)) AND ((mesh* OR dynamesh* OR vitamesh* OR surgimesh*)) AND ((prevent* OR prophyla* OR augment* OR reinforce*)))

Cochrane CENTRAL

(((aort* NEAR/3 aneurysm*) OR aaa):Ab,ti) AND ((mesh* OR dynamesh* OR vitamesh* OR surgimesh*):ab,ti) AND ((prevent* OR prophyla* OR augment* OR reinforce*):ab,ti)

Google scholar

"aortic|aorta aneurysm|aneurysms" mesh|dynamesh|vitamesh|surgimesh preventive|prevention|prophylaxis|prophylactic|augmentation|reinforcement

van den Berg R, *et al. BMJ Open* 2024; 14:e081046. doi: 10.1136/bmjopen-2023-081046
