# Supplementary material for: Protocol for an independent patient data meta-analysis of prophylactic mesh placement for incisional hernia prevention after abdominal aortic aneurysm surgery: a collaborative European Hernia Society project (I-PREVENT-AAA)
Source: BMJ Open. 2024 Apr 16;14(4):e081046. doi: 10.1136/bmjopen-2023-081046 (PMC11029178; doi:10.1136/bmjopen-2023-081046)
Supplement: Author's manuscript [file bmjopen-2023-081046.draft_revisions.pdf]

BMJ Open

BMJ Open is committed to open peer review. As part of this commitment we make the peer review history of every article we publish publicly available.

When an article is published we post the peer reviewers' comments and the authors' responses online. We also post the versions of the paper that were used during peer review. These are the versions that the peer review comments apply to.

The versions of the paper that follow are the versions that were submitted during the peer review process. They are not the versions of record or the final published versions. They should not be cited or distributed as the published version of this manuscript.

BMJ Open is an open access journal and the full, final, typeset and author-corrected version of record of the manuscript is available on our site with no access controls, subscription charges or pay-per-view fees (http://bmjopen.bmj.com).

If you have any questions on BMJ Open's open peer review process please email info.bmjopen@bmj.com

BMJ Open

Protocol for an Independent Patient Data Meta-Analysis of Prophylactic Mesh Placement for Incisional Hernia Prevention (after Abdominal Aortic Aneurysm surgery): A collaborative European Hernia Society project

Journal:	*BMJ Open*
Manuscript ID	bmjopen-2023-081046
Article Type:	Protocol
Date Submitted by the Author:	17-Oct-2023

Complete List of Authors:	van den Berg, Rudolf; Erasmus MC, Surgery; Erasmus University Rotterdam, Medicine<br>den Hartog, Floris; Erasmus MC, Surgery<br>Bali, Christina; University General Hospital of Ioannina, Surgery<br>Matsagkas, Miltiadis; University of Thessaly, Vascular Surgery<br>Bevis, Paul; North Bristol NHS Trust, Vascular Surgery<br>Earnshaw, Jonothan; Gloucestershire Hospitals NHS Foundation Trust, Vascular Surgery<br>Debus, Eike; University Medical Center Hamburg-Eppendorf University Heart & Vascular Center, Vascular medicine<br>Honig, Susanne; Hospital Robert Schuman Kirchberg Hospital, Chirurgie Vasculaire<br>Berrevoet, Frederik; University Hospital Ghent, Department of General and HPB Surgery and Liver Transplantation<br>Detry, Olivier; Universite de Liege, Chirurgie Abdominale & Transplantation<br>Stabilini, Cesare; University of Genoa, Surgical Sciences<br>Muysoms , Filip ; AZ Maria Middelares vzw, Surgery<br>Tanis, Pieter; Erasmus MC, Surgery<br>collaborators, European Hernia Society Prophylactic; European Hernia Society

Keywords:	SURGERY, PREVENTIVE MEDICINE, Adolescent

SCHOLARONE™ Manuscripts

Protocol for an Independent Patient Data Meta-Analysis of Prophylactic Mesh Placement for Incisional Hernia Prevention (after Abdominal Aortic Aneurysm surgery): A collaborative European Hernia Society project

Rudolf van den Berg[1], Floris P.J. Den Hartog[1], Christina Bali[2], Miltiadis Matsagkas[3], Paul M. Bevis[4], Jonothan J. Earnshaw[5], Eike S. Debus[6], Susanne Honig[7], Frederik Berrevoet[8], Olivier Detry[9], Cesare Stabilini[10], Filip E. Muysoms[11], Pieter J. Tanis[1] , European Hernia Society Prophylactic mesh study group collaborators

European Hernia Society Prophylactic mesh study group collaborators:
Holger Diener[6], Tilo Kölbel[6], Wolfgang Reinpold[12], Antonia Zapf[13], Eric Bibiza-Freiwald[13], Vaia K. Georvasili[2]

Corresponding author: Rudolf van den Berg: r.vandenberg.4@erasmusmc.nl, postal address: Dr. Molewaterplein 40, 3015 GD Rotterdam
[1] Department of Surgery, Erasmus University Medical Centre, Rotterdam, Netherlands.
[2] Department of Surgery, University Hospital of Ioannina, Ioannina, Greece.
[3] Department of Vascular Surgery, University of Thessaly, Larissa, Greece.
[4] Department of Vascular Surgery, North Bristol NHS Trust, United Kingdom.
[5] Department of Vascular Surgery, Gloucestershire Health and Care NHS Foundation Trust, United Kingdom.
[6] Department of Vascular Medicine, University Medical Center Hamburg-Eppendorf University Heart & Vascular Center, Eppendorf, Hamburg, Germany.
[7] Department of Vascular Surgery, Hospital Robert Schuman Kirchberg Hospital, Luxembourg.
[8] Department of General and Hepatobiliary Surgery and Liver Transplantation, Ghent University Hospital, Ghent, Belgium.
[9] Department of Abdominal Surgery and Transplantation, CHU Liege, Belgium.
[10] Department of Surgical Sciences, University of Genoa, Genoa, Italy.
[11] Department of Surgery, AZ Maria Middelares Hospital, Ghent, Belgium.
[12] Hamburg Hernia Centre, Department of Hernia and Abdominal Wall Surgery, Helios Mariahilf Hospital Hamburg, Teaching Hospital of the University of Hamburg, Hamburg, Germany
[13] Institut für Medizinische Biometrie und Epidemiologie, Universitätsklinikum Hamburg-Eppendorf, Hamburg, Germany

Funding statement

The European Hernia Society supported open-access publication by subsidizing the article processing fee. The study received no further grant from any funding agency in the public, commercial or not-for-profit sectors.

Category:

Protocol paper for an independent patient data meta-analysis

Competing interests statement

Participating authors of the I-PREVENT study have previously worked on the PRIMA and PRIMAAT trials. These authors will be excluded from doing quality evaluation and data extraction of relevant trials. No further statements of competing interests need to be disclosed.

Data availability statement

Data used in this study is available upon request.

For peer review only

Abstract

Introduction

Incisional hernia (IH) is a prevalent and potentially dangerous complication after abdominal surgery, especially in high-risk groups. Mesh reinforcement of the abdominal wall has been studied as a potential intervention to prevent IHs. Randomised controlled trials (RCTs) have demonstrated that prophylactic mesh reinforcement after abdominal surgery, in general, is effective and safe. In patients with abdominal aortic aneurysm (AAA), prophylactic mesh reinforcement after open repair has not yet been recommended in official guidelines, because of relatively small sample sizes in individual trials. Furthermore, identification of subgroups that benefit most from prophylactic mesh placement requires larger patient numbers. Our primary aim is to evaluate the efficacy and effectiveness of the use of a prophylactic mesh after open AAA surgery for prevention of IH by performing an individual patient data meta-analysis (IPDMA). Secondary aims include evaluation of postoperative complications, pain and quality of life, and identification of potential subgroups that benefit most from prophylactic mesh reinforcement.

Methods and analysis

We will conduct a systematic review to identify RCTs that study prophylactic mesh placement after open AAA surgery. Lead authors of eligible studies will be asked to share individual participant data (IPD). Risk of bias (ROB) for each included study will be assessed using the Cochrane ROB tool. An IPDMA will be performed to evaluate efficacy, with time to IH as primary outcome. Any signs of heterogeneity will be evaluated by Forest plots. Time-to-event analyses are performed using Cox regression analysis, also for evaluation of risk factors.

Ethics and dissemination

No new data will be collected in this study. We will adhere to institutional, national and international regulations regarding the secure and confidential sharing of IPD, addressing ethics as indicated. We will disseminate findings via international conferences, open-source publication in peer-reviewed journals and summaries posted online.

PROSPERO registration number: CRD42022347881.

MESH-terms: Incisional hernia / prevention & control, Surgical Mesh, Suture Techniques, Aortic Aneurysm

Strengths and limitations of this study

- We designed our protocol in collaboration with the European Hernia Society, an internationally recognised organization with experience in procedures for navigating the safe transfer and storage of IPD.
- IPD meta-analyses of randomised clinical trials enhance the ability to handle participant-level and study-level confounding, and increases the power to identify responder subgroups and confounding factors underlying treatment effects.
- A key limitation to undertaking IPD analyses relates to overcoming data-sharing hurdles, and the achievement of our aims will in part depend on the ability to successfully obtain IPD from eligible studies.
- The protocol for this independent patient data meta-analysis was written according to the PRISMA-P guidelines.

For peer review only

Introduction

Incisional hernia (IH) is a type of ventral abdominal wall hernia which occurs in or near the scar of a previous surgical incision. The typical presentation is a visible or palpable bulge which increases in size and visibility when the intra-abdominal pressure (IAP) is raised. Patients with IH are at risk for incarceration, bowel obstruction or strangulation, with an ischemic bowel and emergency surgery with potential bowel resection and ostomy formation as a result[1,2]. Patients' daily functioning and social life can be affected, and serious mental issues can arise due to a changed body image[3-5]. IH repair has a big economic burden due to its prevalence and costs[6]. The only curative therapy is surgical reconstruction with mesh implantation, which can be very extensive surgery depending on hernia characteristics such as diameter and location of the hernia.

Patients who undergo elective abdominal or pelvic surgery, where a median laparotomy is performed, have an up to 30% risk of IH formation. Typically, IH becomes evident within two years after surgery[7-9]. In high-risk groups or after emergency surgery, the IH incidence can become as high as 69%[10-14]. High risk groups are patients with a high Body Mass Index (BMI, $\geq$ 27 kg/m^2) or patients who underwent open repair of an abdominal aortic aneurysm (AAA)[15]. Patients with an AAA might have an underlying connective tissue disorder and it is hypothesised that this impairment also plays a role in the pathogenesis of IH.

Prevention of IH formation is a key issue in abdominal wall research. Different incision directions and locations, suture techniques and prophylactic reinforcement with mesh have been considered, with mixed outcomes. Conventional meta-analyses (MA) of randomized controlled trials (RCTs) have demonstrated that, in general, prophylactic mesh augmentation (PMA) after midline laparotomy is effective, safe and cost-effective[16]. However, due to problems with study design and sample size, the strength of recommendations for actually incorporating PMA in daily practice for elective midline laparotomies is weak[16]. PMA has also been studied in high-risk groups, albeit in a much smaller number of studies[3]. For AAA specifically, the European Society for Vascular Surgery (ESVS) guideline states that PMA after open AAA repair 'may be considered' (a class IIb recommendation, level of evidence A)[14]. This recommendation is based on one of the latest meta-analysis (Table 1)[17]. Long-term results of two RCTs in that analysis, the PRIMA and PRIMAAT trial, have not yet been included in any meta-analysis[18,19].

To date, no study on this topic has pooled individual participant data (IPD) across studies. An IPD meta-analysis (IPDMA) evaluates raw units of data rather than aggregated study-level data, and is thus a more robust approach to evaluating treatment effect modifiers and mediators. Compared with traditional study-level MAs, IPDMAs enhance the ability to handle participant-level and study-level confounding, provide more complete analyses of time-to-event outcomes, and increase the power to identify responder subgroups and mechanisms underlying treatment effects. The outcomes resulting from using such an approach may, therefore, be more reliable and generalizable.

By combining the IPD of relevant RCTs together and performing statistical analyses on the combined, patient-level data, we strive to raise the level of evidence regarding mesh prophylaxis for IH prevention after open AAA repair and to help identify those who will benefit most from this procedure. This can only be achieved through international collaboration. Despite the growing recognition of the ethical and scientific importance of data sharing and scientific transparency, one of the biggest challenges in undertaking IPD analyses relates to overcoming data-sharing hurdles. Barriers range from successfully reaching original study authors; willingness or ability of authors

to share data; and international ethics and regulations issues. For this study, collaboration will be initiated through the European Hernia Society (EHS). The EHS is an internationally recognized organization in the field of hernia surgery, and it has appointed a steering committee to oversee this IPDMA.

Current knowledge/data

Study	Types of surgery	Risk-ratio incidence of IH	Risk-ratio of reoperation for IH	Risk-ratio post- operative seroma	Risk ratio post-operative SSI
Indrakusuma et al. (2018)[17]	AAA open repair surgery	0.27 (0.11-0.66)	0.23 (0.05-1.05)	x	x
Aiolfi et al. (2022)[20]	All midline incisions	0.38 (0.24-0.58)	x	2.05 (1.35-3.13)	1.17 (0.82-1.67)
Jairam et al. (2020)[3]	All elective midline incisions	0.35 (0.21-0.57)	x	Onlay 2,23 (1,10 - 4,52)<br>Retromuscular 1,67 (0,81 – 3,47)	Onlay 1,67 (0,81 – 3,47)<br>Retromuscular 0,28 (0,10 – 0,82)

Table 1: Most recently published summary data of incisional hernia prevention by prophylactic mesh placement.

Aims

We aim to conduct a systematic review and IPDMA of RCTs, to evaluate the effectiveness of the use of a prophylactic mesh after open AAA surgery for prevention of IH. The time to IH occurrence during long-term follow-up as primary outcome will be compared between prophylactic mesh reinforcement and primary sutured closure. Our secondary aims are to evaluate differences in postoperative complications, pain, and quality of life, if documented in the original trial, and to identify potential subgroups of patients who will benefit most from prophylactic mesh reinforcement after open AAA surgery. The results of this study will support recommendations in future guideline updates, and they will directly inform clinicians regarding abdominal wall closure after open AAA repair. This will translate into benefit for those who will undergo AAA repair. Ultimately, reducing the incidence of IH after AAA repair is a socially responsible goal, as it will also result in reduced societal healthcare costs.

Methods and analysis

The basic study protocol was approved by the EHS scientific committee. Subsequently, it was submitted for registration to the International Prospective Register of Systematic Reviews (PROSPERO) (registration number CRD42022347881). It formed the basis for the present, detailed protocol, which was written in accordance with the Preferred Reporting Items for Systematic Reviews and Meta-Analyses Protocols (PRISMA) statement and PRISMA IPD (PRISMA-IPD) guidelines. Data transfer methods, developed in collaboration with the Erasmus MC data transfer office (DTO) and approved by the EHS, will guide the secure transfer and responsible use of IPD, adhering to current European data-sharing regulations.

Study identification

A literature search will be performed in the following databases: Cochrane Central Register of Controlled Trials (CENTRAL); MEDLINE Ovid (1946 onwards); Embase (1980 onwards); Web of Science Core Collection (1975 onwards) and Google Scholar. The search strategy will be tailor-made, by the investigators, together with an experienced, professional librarian from the Erasmus MC Medical Library.

Data procurement

For all identified studies, we will contact the corresponding author by email. If a current email address cannot be found or the author does not respond (up to three attempts), we will attempt to reach them by other means (phone,

For peer review only

post, contact institution or any other means of contact that are available). Where IPD are available and authors or institutions are willing to share data, a data delivery agreement (DDA) will be drafted by both parties. A template DDA has been prepared for this study and it will be reasonably adapted if authors see the need to make changes to it, after which it will be signed. Dutch ethics regulations do not require explicit ethical approval for conducting IPDMAs. However, where local ethics regulations require it, ethics approval will be sought prior to sharing data. Pseudonymised or anonymised data sets (all formats are acceptable, e.g., SPSS, Excel) and related data dictionaries will then be transferred and stored securely in a database at Erasmus MC, for use only as agreed on in the DDA. One original study investigator (first or senior author, at the discretion of the data owner) will be invited to be a co-author of the project if they are willing to assume responsibilities that meet authorship guidelines, as also stated in the DDA.

Data processing and validation

We will convert all data sets to a common format, combine data sets with a new variable identifying original trial and harmonize variables. Data checking will include evaluating baseline characteristics and results of comparisons for our main outcomes against results reported in original publications. We will also check for balancing of baseline participant characteristics in each treatment arm, and evaluate the extent to which all randomized participants in the IPD datasets have been included in study analyses. Authors will be consulted in the case of any inconsistencies or discrepancies. In cases where discrepancies cannot be resolved, we will (on a case-by-case basis) either conduct a sensitivity analysis with that study removed, or we will exclude the study from our analysis altogether.

Two independent investigators will parse data from all included published studies. From each study, we will extract the following data: country of study; funding source; study design; sample size; target population; inclusion/exclusion criteria; participant characteristics (age, sex, BMI, history of injury or surgery, comorbidities, medication use); type and context of intervention; AAA characteristics; pain and quality of life pre–post as available. For all patient-reported outcomes, we will extract the recall period in addition to the outcome. Where IPD are available, we will conduct all analyses using IPD instead of aggregate data, following data consistency checks described above.

Study quality assessment

Two investigators will independently evaluate risk of bias (ROB) for each included study using the Cochrane ROB tool, and disagreements will be resolved by a third investigator. Any authors involved in any included trial will not extract data from or assess the risk of bias in those trials. Duplicate publications will be identified to evaluate the trials and all available data simultaneously to maximize data extraction and correct bias assessment. The Cochrane ROB considers five domains of possible bias: randomization; deviations from intended interventions; missing outcome data; measurement of the outcome and selection of the reported results. For each domain, ROB is rated as low, some concerns or high. The overall study will be considered to be of low ROB if all five domains are rated as low ROB, and high overall ROB if at least one domain is rated as high ROB or if some concerns are identified in multiple domains. We will consult authors of the original publications in the event of inadequate reporting or inconsistencies. If indicated, we will email the authors to request data that may not have been sufficiently included in the primary publication.

The following trial-related data will be extracted:

For peer review only

- Trial characteristics: bias risk components, trial design, period and number of sites, countries where the trial was conducted, number of intervention arms, length of follow-up and inclusion and exclusion criteria.
- Participant characteristics and comorbidities: number of randomized participants, analysed participants, participants lost to follow-up, mean age, age range, sex ratio, specific patient-based inclusion criteria, treatment characteristics (e.g., operating time).

Assessment of heterogeneity

Forest plots will be constructed to visualise and assess any signs of heterogeneity. Statistical heterogeneity will be assessed using the Chi-square test (threshold $p < .10$), the quantities of heterogeneity will be measured with the I^2 statistic, and possible heterogeneity will be assessed with relevant subgroup analyses.

All eligible patients from included RCTs will be included for final analysis if meeting the following criteria: adults (18 years or older), diagnosed with AAA using any common method (eg, radiographs, CT, clinical criteria, diagnosis by a healthcare professional). Additionally, inclusion criteria from included RCTs will be evaluated and the criteria of the IPDMA will be amended if required. A potential subject who meets any of the following criteria will be excluded from final analysis: emergency surgery or the presence of a mesh in the abdominal wall on the midline from previous hernia repair. Additionally, exclusion criteria from included RCTs will be evaluated and the criteria of the IPDMA will be amended accordingly.

Sample size calculations stated in the included studies will be assessed. New power calculations will be performed for subgroup analyses that are performed on IPD. A one-stage meta-analysis of IPD will be performed on the data received from the different included studies, which were identified through the literature search. We will conduct time-to-event analysis for all included patients using Cox regression analysis with trial and centre (nested under trial) as cluster terms to compare groups with and without the placement of the prophylactic mesh by the use of the hazard ratio and the corresponding two-sided 95% confidence interval[21]. Risk factors will be evaluated using Cox-regression analysis. Comparison of categorical and continuous variables between groups will be performed using mixed logistic regression analysis with, but not limited to, baseline value, age, gender, and operation indication as possible covariates and trial and centre (nested under trial) as random effects.

Missing data

To avoid bias induced by ignoring missing data in clinical research, it is widely acknowledged that imputation techniques can be considered to replace missing values. We anticipate that the proportion of missing values for the primary and secondary outcomes will be less than 5%, in trials that documented these parameters, and therefore we will consider imputation. For partially missing data, traditional multiple imputation techniques will be performed per individual dataset, if not yet done by the researchers from the study. But also, if the proportion of missing values in relation to the total dataset is reasonably small allowing for the construction of a robust imputation model. However, in a secondary analysis, we will consider using multiple imputation and/or best-worst and worst-best case scenarios if we can’t ignore missing data. We will describe the proportion of missing values for each dataset included in the IPDMA.

For peer review only - http://bmjopen.bmj.com/site/about/guidelines.xhtml

Treatment efficacy

To evaluate treatment efficacy, we want to employ a one-step meta-analysis. This will result in harmonising all data in one large dataset and analysing pooled outcome data of all included patients in the different RCT's, controlling for stratification per centre (indicated by an additional unique covariate for each of the different trials). We will analyse the effect of the treatment by intention to treat, regardless of the methods used in the original study. If a one-stage meta-analysis is not feasible, we will conduct a two-stage meta-analysis where we will first analyse each trial separately, and then pool results across trials. In step 1, within each trial, we will evaluate the effect of assigned intervention by intention to treat, regardless of method used in the original study. If study heterogeneity prevents us from harmonising data, then we will navigate this using a statistical approach based on available data. This will likely involve transforming data into standardised means differences or applying proportion of maximum scaling methods.

In studies where we are unable to obtain IPD, we will extract aggregate data from published manuscripts as they are reported in the published articles. Similar models will be performed for secondary outcomes as data permit. In cases of dichotomous outcomes, we will perform binary modelling and report effect sizes as relative risk (RR, 95% CI).

In step 2, we will perform random effects meta-analysis employing restricted maximum likelihood. We will report study heterogeneity as I^2 and τ^2. In cases of notable heterogeneity (I^2 >50%), we will consider possible sources such as study design, treatment duration, comparison treatment, treatment adherence or study quality. We will then consider performing meta-regression, subgroup analysis or sensitivity analyses to explain or account for these potential sources of heterogeneity. We will pool results of studies both with and without IPD data after verifying that effect sizes of IPD studies do not differ from non-IPD studies.

Treatment effect-modifier analyses

We will conduct treatment effect-modifier analyses to identify subgroups of individuals that will undergo open-AAA operation who benefit most from the placement of the prophylactic mesh by including interaction terms between subgroup and treatment group in the corresponding regression analyses. We have proposed several subgroup characteristics that we hypothesize may modify the effect of the prophylactic mesh on our main outcome (IH formation), based on expert opinion. These proposed subgroups include the following baseline characteristics: (1) BMI score (Patients with a higher BMI are at a higher risk for the development of an IH); (2) Primary fascial closure with different SL.WL ratio's (A higher SL/WL ratio results in less IH's and therefore the use of different SL/WL ratio's might result in wrong conclusions and/or recommendations); (3) Patients with connective tissue disorders (Can be associated with the formation of the AAA and also the healing of the abdominal wall and therefore the formation of an IH).

Patient and Public Involvement

No patient involvement was sought for the development of the protocol for this IPDMA.

Ethics and dissemination

No new data will be collected in this study. We will adhere to institutional, national and international regulations regarding the secure and confidential sharing of IPD, addressing ethics as indicated. We intend to publish the IPDMA in a peer reviewed journal.

For peer review only

Handling and storage of data and documents

Patient data from the participating centres where the RCT's were held, will be anonymized, and transferred via encrypted and secure data transfer. Before data transfer, a data delivery agreement will be signed by both parties. The EHS will handle and store data as an independent party. Only the assigned researcher in the Erasmus MC will have access to the data. No sponsor is present for the study.

Acknowledgments

We thank dr. W.M. Bramer at the ErasmusMC University Medical Centre Medical Library for assisting us with developing our search terms and managing our search.

Author contributions

All authors were involved in the study design and all will contribute to the interpretation of the results. RB contacted the potential data deliverers, will coordinate the data collection, and perform/ supervise the data analyses. RB wrote the manuscript together with FD and PT. RB and FD will have full access to the study data. All authors approved the final manuscript.

The planned search strategy

The search that is planned to be used on Embase is:

('abdominal aortic aneurysm'/exp OR 'aortic aneurysm'/de OR ((aneurysm/de OR 'aneurysm surgery'/de) AND 'abdominal aorta'/de) OR ((aort* NEAR/3 aneurysm*) OR aaa):Ab,ti) AND ('surgical mesh'/exp OR (mesh* OR dynamesh* OR vitamesh* OR surgimesh*):ab,ti) AND (prophylaxis/de OR prevention/de OR prevention:lnk OR (prevent* OR prophyla* OR augment* OR reinforce*):ab,ti)

Word count: 2764

For peer review only

References

1. Azin A, Hirpara D, Jackson T, Okrainec A, Elnahas A, Chadi SA, Quereshy FA. Emergency laparoscopic and open repair of incarcerated ventral hernias: a multi-institutional comparative analysis with coarsened exact matching. Surg Endosc. 2019 Sep;33(9):2812-2820.

2. Dadashzadeh ER, Huckaby LV, Handzel R, Hossain MS, Sanin GD, Anto VP, Bou-Samra P, Moses JB, Cai S, Phelos HM, Simmons RL, Rosengart MR, van der Windt DJ. The Risk of Incarceration During Nonoperative Management of Incisional Hernias: A Population-based Analysis of 30,998 Patients. Ann Surg. 2022 Feb 1;275(2):e488-e495.

3. Jairam AP, Lopez-Cano M, Garcia-Alamino JM, Pereira JA, Timmermans L, Jeekel J, et al. Prevention of incisional hernia after midline laparotomy with prophylactic mesh reinforcement: a meta-analysis and trial sequential analysis. BJS Open. 2020;4(3):357-68.

4. van Ramshorst GH, Eker HH, Hop WC, Jeekel J, Lange JF. Impact of incisional hernia on health-related quality of life and body image: a prospective cohort study. Am J Surg. 2012;204(2):144-50.

5. van Ramshorst GH, Eker HH, van der Voet JA, Jeekel J, Lange JF. Long-term outcome study in patients with abdominal wound dehiscence: a comparative study on quality of life, body image, and incisional hernia. J Gastrointest Surg. 2013;17(8):1477-84.

6. Gillion JF, Sanders D, Miserez M, Muysoms F. The economic burden of incisional ventral hernia repair: a multicentric cost analysis. Hernia. 2016;20(6):819-30.

7. Carney MJ, Weissler JM, Fox JP, Tecce MG, Hsu JY, Fischer JP. Trends in open abdominal surgery in the United States-Observations from 9,950,759 discharges using the 2009-2013 National Inpatient Sample (NIS) datasets. Am J Surg. 2017;214(2):287-92.

8. Rahbari NN, Knebel P, Diener MK, Seidlmayer C, Ridwelski K, Stoltzing H, et al. Current practice of abdominal wall closure in elective surgery - Is there any consensus? BMC Surg. 2009;9:8.

9. Valeriy Shubinets JPF, Michael A Lanni, Michael G Tecce, Eric M Pauli, William W Hope, Stephen J Kovach, John P Fischer. Incisional Hernia in the United States: Trends in Hospital Encounters and Corresponding Healthcare Charges. Am Surg. 2018;1:118-25.

10. Abo-Ryia MH, El-Khadrawy OH, Abd-Allah HS. Prophylactic preperitoneal mesh placement in open bariatric surgery: a guard against incisional hernia development. Obes Surg. 2013;23(10):1571-4.

11. Gutierrez de la Pena C, Medina Achirica C, Dominguez-Adame E, Medina Diez J. Primary closure of laparotomies with high risk of incisional hernia using prosthetic material: analysis of usefulness. Hernia. 2003;7(3):134-6.

12. Pans A, Elen P, Dewe W, Desaive C. Long-term results of polyglactin mesh for the prevention of incisional hernias in obese patients. World J Surg. 1998;22(5):479-82; discussion 82-3.

13. Strzelczyk JM, Szymanski D, Nowicki ME, Wilczynski W, Gaszynski T, Czupryniak L. Randomized clinical trial of postoperative hernia prophylaxis in open bariatric surgery. Br J Surg. 2006;93(11):1347-50.

14. Wanhainen A, Verzini F, Van Herzeele I, Allaire E, Bown M, Cohnert T, et al. Editor's Choice - European Society for Vascular Surgery (ESVS) 2019 Clinical Practice Guidelines on the Management of Abdominal Aorto-iliac Artery Aneurysms. Eur J Vasc Endovasc Surg. 2019;57(1):8-93.

For peer review only

15. Jairam AP, Timmermans L, Eker HH, Pierik R, van Klaveren D, Steyerberg EW, et al. Prevention of incisional hernia with prophylactic onlay and sublay mesh reinforcement versus primary suture only in midline laparotomies (PRIMA): 2-year follow-up of a multicentre, double-blind, randomised controlled trial. Lancet. 2017;390(10094):567-76.

16. Deerenberg EB, Henriksen NA, Antoniou GA, Antoniou SA, Bramer WM, Fischer JP, et al. Updated guideline for closure of abdominal wall incisions from the European and American Hernia Societies. Br J Surg. 2022.

17. Indrakusuma R, Jalalzadeh H, van der Meij JE, Balm R, Koelemay MJW. Prophylactic Mesh Reinforcement versus Sutured Closure to Prevent Incisional Hernias after Open Abdominal Aortic Aneurysm Repair via Midline Laparotomy: A Systematic Review and Meta-Analysis. Eur J Vasc Endovasc Surg. 2018;56(1):120-8.

18. Dewulf M, Muysoms F, Vierendeels T, Huyghe M, Miserez M, Ruppert M, et al. Prevention of Incisional Hernias by Prophylactic Mesh-augmented Reinforcement of Midline Laparotomies for Abdominal Aortic Aneurysm Treatment: Five-year Follow-up of a Randomized Controlled Trial. Ann Surg. 2022;276(4):e217-e22.

19. Jairam AP, Timmermans L, Eker HH, Pierik RE, van Klaveren D, Steyerberg EW, et al. Long Term Follow-up PRIMA Trial. [Updated 2022 Jul 18]. Available from: https://onderzoekmetmensen.nl/nl/trial/22792.

20. Aiolfi, A., et al. Prophylactic mesh reinforcement for midline incisional hernia prevention: systematic review and updated meta-analysis of randomized controlled trials. Hernia. 2023 May; 27(2): 213-224.

21. de Jong VMT, Moons KGM, Riley RD, Tudur Smith C, Marson AG, Eijkemans MJC, Debray TPA. Individual participant data meta-analysis of intervention studies with time-to-event outcomes: A review of the methodology and an applied example. Res Synth Methods. 2020 Mar;11(2):148-168.

For peer review only

RESEARCH METHODS & REPORTING

Table 2| PRISMA-P (preferred reporting items for systematic review and meta-analysis protocols) 2015 checklist: recommended items to address in a systematic review protocol

Section and topic	Item No	Checklist item
**Administrative information**		
Title:		
Identification	1a	Identify the report as a protocol of a systematic review V
Update	1b	If the protocol is for an update of a previous systematic review, identify as such V
Registration	2	If registered, provide the name of the registry (such as PROSPERO) and registration number V
Authors:		
Contact	3a	Provide name, institutional affiliation, e-mail address of all protocol authors; provide physical mailing address of corresponding author V
Contributions	3b	Describe contributions of protocol authors and identify the guarantor of the review V
Amendments	4	If the protocol represents an amendment of a previously completed or published protocol, identify as such and list changes; otherwise, state plan for documenting important protocol amendments V
Support:		
Sources	5a	Indicate sources of financial or other support for the review V
Sponsor	5b	Provide name for the review funder and/or sponsor V
Role of sponsor or funder	5c	Describe roles of funder(s), sponsor(s), and/or institution(s), if any, in developing the protocol V
**Introduction**		
Rationale	6	Describe the rationale for the review in the context of what is already known V
Objectives	7	Provide an explicit statement of the question(s) the review will address with reference to participants, interventions, comparators, and outcomes (PICO) V
**Methods**		
Eligibility criteria	8	Specify the study characteristics (such as PICO, study design, setting, time frame) and report characteristics (such as years considered, language, publication status) to be used as criteria for eligibility for the review V
Information sources	9	Describe all intended information sources (such as electronic databases, contact with study authors, trial registers or other grey literature sources) with planned dates of coverage V
Search strategy	10	Present draft of search strategy to be used for at least one electronic database, including planned limits, such that it could be repeated V
Study records:		
Data management	11a	Describe the mechanism(s) that will be used to manage records and data throughout the review V
Selection process	11b	State the process that will be used for selecting studies (such as two independent reviewers) through each phase of the review (that is, screening, eligibility and inclusion in meta-analysis) V
Data collection process	11c	Describe planned method of extracting data from reports (such as piloting forms, done independently, in duplicate), any processes for obtaining and confirming data from investigators V
Data items	12	List and define all variables for which data will be sought (such as PICO items, funding sources), any pre-planned data assumptions and simplifications V
Outcomes and prioritization	13	List and define all outcomes for which data will be sought, including prioritization of main and additional outcomes, with rationale V
Risk of bias in individual studies	14	Describe anticipated methods for assessing risk of bias of individual studies, including whether this will be done at the outcome or study level, or both; state how this information will be used in data synthesis V
Data synthesis	15a	Describe criteria under which study data will be quantitatively synthesised V
	15b	If data are appropriate for quantitative synthesis, describe planned summary measures, methods of handling data and methods of combining data from studies, including any planned exploration of consistency (such as I^2, Kendall's τ) V
	15c	Describe any proposed additional analyses (such as sensitivity or subgroup analyses, meta-regression) V
	15d	If quantitative synthesis is not appropriate, describe the type of summary planned V
Meta-bias(es)	16	Specify any planned assessment of meta-bias(es) (such as publication bias across studies, selective reporting within studies) V
Confidence in cumulative evidence	17	Describe how the strength of the body of evidence will be assessed (such as GRADE) V

RESEARCH METHODS & REPORTING

Table 2| PRISMA-P (preferred reporting items for systematic review and meta-analysis protocols) 2015 checklist: recommended items to address in a systematic review protocol

Section and topic	Item No	Checklist item
**Administrative information**		
Title:		
Identification	1a	Identify the report as a protocol of a systematic review V
Update	1b	If the protocol is for an update of a previous systematic review, identify as such V
Registration	2	If registered, provide the name of the registry (such as PROSPERO) and registration number V
Authors:		
Contact	3a	Provide name, institutional affiliation, e-mail address of all protocol authors; provide physical mailing address of corresponding author V
Contributions	3b	Describe contributions of protocol authors and identify the guarantor of the review V
Amendments	4	If the protocol represents an amendment of a previously completed or published protocol, identify as such and list changes; otherwise, state plan for documenting important protocol amendments V
Support:		
Sources	5a	Indicate sources of financial or other support for the review V
Sponsor	5b	Provide name for the review funder and/or sponsor V
Role of sponsor or funder	5c	Describe roles of funder(s), sponsor(s), and/or institution(s), if any, in developing the protocol V
**Introduction**		
Rationale	6	Describe the rationale for the review in the context of what is already known V
Objectives	7	Provide an explicit statement of the question(s) the review will address with reference to participants, interventions, comparators, and outcomes (PICO) V
**Methods**		
Eligibility criteria	8	Specify the study characteristics (such as PICO, study design, setting, time frame) and report characteristics (such as years considered, language, publication status) to be used as criteria for eligibility for the review V
Information sources	9	Describe all intended information sources (such as electronic databases, contact with study authors, trial registers or other grey literature sources) with planned dates of coverage V
Search strategy	10	Present draft of search strategy to be used for at least one electronic database, including planned limits, such that it could be repeated V
Study records:		
Data management	11a	Describe the mechanism(s) that will be used to manage records and data throughout the review V
Selection process	11b	State the process that will be used for selecting studies (such as two independent reviewers) through each phase of the review (that is, screening, eligibility and inclusion in meta-analysis) V
Data collection process	11c	Describe planned method of extracting data from reports (such as piloting forms, done independently, in duplicate), any processes for obtaining and confirming data from investigators V
Data items	12	List and define all variables for which data will be sought (such as PICO items, funding sources), any pre-planned data assumptions and simplifications V
Outcomes and prioritization	13	List and define all outcomes for which data will be sought, including prioritization of main and additional outcomes, with rationale V
Risk of bias in individual studies	14	Describe anticipated methods for assessing risk of bias of individual studies, including whether this will be done at the outcome or study level, or both; state how this information will be used in data synthesis V
Data synthesis	15a	Describe criteria under which study data will be quantitatively synthesised V
	15b	If data are appropriate for quantitative synthesis, describe planned summary measures, methods of handling data and methods of combining data from studies, including any planned exploration of consistency (such as I^2, Kendall's τ) V
	15c	Describe any proposed additional analyses (such as sensitivity or subgroup analyses, meta-regression) V
	15d	If quantitative synthesis is not appropriate, describe the type of summary planned V
Meta-bias(es)	16	Specify any planned assessment of meta-bias(es) (such as publication bias across studies, selective reporting within studies) V
Confidence in cumulative evidence	17	Describe how the strength of the body of evidence will be assessed (such as GRADE) V

BMJ Open

Protocol for an Independent Patient Data Meta-Analysis of Prophylactic Mesh Placement for Incisional Hernia Prevention (after Abdominal Aortic Aneurysm surgery): A collaborative European Hernia Society project

Journal:	*BMJ Open*
Manuscript ID	bmjopen-2023-081046.R1
Article Type:	Protocol
Date Submitted by the Author:	21-Feb-2024

Complete List of Authors:	van den Berg, Rudolf; Erasmus MC, Surgery; Erasmus University Rotterdam, Medicine<br>den Hartog, Floris; Erasmus MC, Surgery<br>Bali, Christina; University General Hospital of Ioannina, Surgery<br>Matsagkas, Miltiadis; University of Thessaly, Vascular Surgery<br>Bevis, Paul; North Bristol NHS Trust, Vascular Surgery<br>Earnshaw, Jonothan; Gloucestershire Hospitals NHS Foundation Trust, Vascular Surgery<br>Debus, Eike; University Medical Center Hamburg-Eppendorf University Heart & Vascular Center, Vascular medicine<br>Honig, Susanne; Hospital Robert Schuman Kirchberg Hospital, Chirurgie Vasculaire<br>Berrevoet, Frederik; University Hospital Ghent, Department of General and HPB Surgery and Liver Transplantation<br>Detry, Olivier; Universite de Liege, Chirurgie Abdominale & Transplantation<br>Stabilini, Cesare; University of Genoa, Surgical Sciences<br>Muysoms , Filip ; AZ Maria Middelares vzw, Surgery<br>Tanis, Pieter; Erasmus MC, Surgery<br>Prophylactic Mesh Study Group Collaborators, European Hernia Society ; -

**Primary Subject Heading**:	Surgery
Secondary Subject Heading:	Surgery, Epidemiology
Keywords:	SURGERY, PREVENTIVE MEDICINE, Adolescent

SCHOLARONE™ Manuscripts

Protocol for an Independent Patient Data Meta-Analysis of Prophylactic Mesh Placement for Incisional Hernia Prevention (after Abdominal Aortic Aneurysm surgery): A collaborative European Hernia Society project

Rudolf van den Berg[1], Floris P.J. Den Hartog[1], Christina Bali[2], Miltiadis Matsagkas[3], Paul M. Bevis[4], Jonothan J. Earnshaw[5], Eike S. Debus[6], Susanne Honig[7], Frederik Berrevoet[8], Olivier Detry[9], Cesare Stabilini[10], Filip E. Muysoms[11], Pieter J. Tanis[1] , European Hernia Society Prophylactic mesh study group collaborators

European Hernia Society Prophylactic mesh study group collaborators:
Holger Diener[6], Tilo Kölbel[6], Wolfgang Reinpold[12], Antonia Zapf[13], Eric Bibiza-Freiwald[13], Vaia K. Georvasili[2]

Corresponding author: Rudolf van den Berg: r.vandenberg.4@erasmusmc.nl, postal address: Dr. Molewaterplein 40, 3015 GD Rotterdam

[1] Department of Surgery, Erasmus University Medical Centre, Rotterdam, Netherlands.
[2] Department of Surgery, University Hospital of Ioannina, Ioannina, Greece.
[3] Department of Vascular Surgery, University of Thessaly, Larissa, Greece.
[4] Department of Vascular Surgery, North Bristol NHS Trust, United Kingdom.
[5] Department of Vascular Surgery, Gloucestershire Hospitals NHS Foundation Trust, UK.
[6] Department of Vascular Medicine, University Medical Center Hamburg-Eppendorf University Heart & Vascular Center, Eppendorf, Hamburg, Germany.
[7] Department of Vascular Surgery, Hospital Robert Schuman Kirchberg Hospital, Luxembourg.
[8] Department of General and Hepatobiliary Surgery and Liver Transplantation, Ghent University Hospital, Ghent, Belgium.
[9] Department of Abdominal Surgery and Transplantation, Division of Abdominal Wall Surgery, CHU Liege, University of Liege, Liege, Belgium.
[10] Department of Surgical Sciences, University of Genoa, Genoa, Italy.
[11] Department of Surgery, AZ Maria Middelares Hospital, Ghent, Belgium.
[12] Hamburg Hernia Centre, Department of Hernia and Abdominal Wall Surgery, Helios Mariahilf Hospital Hamburg, Teaching Hospital of the University of Hamburg, Hamburg, Germany
[13] Institute of Medical Biometry and Epidemiology, University Medical Center Hamburg-Eppendorf, Hamburg, Germany

Funding statement

The study received no grant from any funding agency in the public, commercial or not-for-profit sectors.

Category:

Protocol paper for an independent patient data meta-analysis

Competing interests statement

Participating authors of the I-PREVENT study have previously worked on the PRIMA and PRIMAAT trials. These authors will be excluded from doing quality evaluation and data extraction of relevant trials. No further statements of competing interests need to be disclosed.

Data availability statement

Data used in this study is available upon request.

For peer review only

Abstract

Introduction

Incisional hernia (IH) is a prevalent and potentially dangerous complication after abdominal surgery, especially in high-risk groups. Mesh reinforcement of the abdominal wall has been studied as a potential intervention to prevent IHs. Randomised controlled trials (RCTs) have demonstrated that prophylactic mesh reinforcement after abdominal surgery, in general, is effective and safe. In patients with abdominal aortic aneurysm (AAA), prophylactic mesh reinforcement after open repair has not yet been recommended in official guidelines, because of relatively small sample sizes in individual trials. Furthermore, identification of subgroups that benefit most from prophylactic mesh placement requires larger patient numbers. Our primary aim is to evaluate the efficacy and effectiveness of the use of a prophylactic mesh after open AAA surgery for prevention of IH by performing an individual patient data meta-analysis (IPDMA). Secondary aims include evaluation of postoperative complications, pain and quality of life, and identification of potential subgroups that benefit most from prophylactic mesh reinforcement.

Methods and analysis

We will conduct a systematic review to identify RCTs that study prophylactic mesh placement after open AAA surgery. Cochrane Central Register of Controlled Trials; MEDLINE Ovid; Embase; Web of Science Core Collection and Google Scholar will be searched onwards from date of inception. RCTs must directly compare primary sutured closure with mesh closure in adult patients that undergo open AAA surgery, Lead authors of eligible studies will be asked to share individual participant data (IPD). Risk of bias (ROB) for each included study will be assessed using the Cochrane ROB tool. An IPDMA will be performed to evaluate efficacy, with IH rate as primary outcome. Any signs of heterogeneity will be evaluated by Forest plots. Time-to-event analyses are performed using Cox regression analysis, also for evaluation of risk factors.

Ethics and dissemination

No new data will be collected in this study. We will adhere to institutional, national and international regulations regarding the secure and confidential sharing of IPD, addressing ethics as indicated. We will disseminate findings via international conferences, open-source publication in peer-reviewed journals and summaries posted online.

PROSPERO registration number: CRD42022347881.

MESH-terms: Incisional hernia / prevention & control, Surgical Mesh, Suture Techniques, Aortic Aneurysm

Strengths and limitations of this study

- We designed our protocol in collaboration with the European Hernia Society, an internationally recognised organization with experience in procedures for navigating the safe transfer and storage of IPD.
- IPD meta-analyses of randomised clinical trials enhance the ability to handle participant-level and study-level confounding, and increases the power to identify responder subgroups and confounding factors underlying treatment effects.
- A key limitation to undertaking IPD analyses relates to overcoming data-sharing hurdles, and the achievement of our aims will in part depend on the ability to successfully obtain IPD from eligible studies.
- The protocol for this independent patient data meta-analysis was written according to the PRISMA-P guidelines.

For peer review only

Introduction

Incisional hernia (IH) is a type of ventral abdominal wall hernia which occurs in or near the scar of a previous surgical incision. The typical presentation is a visible or palpable bulge which increases in size and visibility when the intra-abdominal pressure (IAP) is raised. Patients with IH are at risk for incarceration, bowel obstruction or strangulation, with an ischemic bowel and emergency surgery with potential bowel resection and ostomy formation as a result [1-2]. Patients' daily functioning and social life can be affected, and serious mental issues can arise due to a changed body image [3-5]. IH repair has a big economic burden due to its prevalence and costs [6]. The only curative therapy is surgical reconstruction with mesh implantation, which can be very extensive surgery depending on hernia characteristics such as diameter and location of the hernia.

Patients who undergo elective abdominal or pelvic surgery, where a median laparotomy is performed, have an up to 30% risk of IH formation. Typically, IH becomes evident within two years after surgery [7-9]. In high-risk groups or after emergency surgery, the IH incidence can become as high as 69% [10-14]. High risk groups are patients with a high Body Mass Index (BMI, $\geq$ 27 kg/m^2) or patients who underwent open repair of an abdominal aortic aneurysm (AAA) [15]. Patients with an AAA might have an underlying connective tissue disorder and it is hypothesised that this impairment also plays a role in the pathogenesis of IH.

Prevention of IH formation is a key issue in abdominal wall research. Different incision directions and locations, suture techniques and prophylactic reinforcement with mesh have been considered, with mixed outcomes. Conventional meta-analyses (MA) of randomized controlled trials (RCTs) have demonstrated that, in general, prophylactic mesh augmentation (PMA) after midline laparotomy is effective, safe and cost-effective [16]. However, due to problems with study design and sample size, the strength of recommendations for actually incorporating PMA in daily practice for elective midline laparotomies is weak [16]. PMA has also been studied in high-risk groups, albeit in a much smaller number of studies [3]. For AAA specifically, the European Society for Vascular Surgery (ESVS) guideline states that PMA after open AAA repair 'may be considered' (a class IIb recommendation, level of evidence A) [14]. This recommendation is based on one of the latest meta-analysis (Table 1) [17]. Long-term results of two RCTs in that analysis, the PRIMA and PRIMAAT trial, have not yet been included in any meta-analysis [18,19].

To date, no study on this topic has pooled individual participant data (IPD) across studies. An IPD meta-analysis (IPDMA) evaluates raw units of data rather than aggregated study-level data, and is thus a more robust approach to evaluating treatment effect modifiers and mediators. Compared with traditional study-level MAs, IPDMAs enhance the ability to handle participant-level and study-level confounding, provide more complete analyses of time-to-event outcomes, and increase the power to identify responder subgroups and mechanisms underlying treatment effects. The outcomes resulting from using such an approach may, therefore, be more reliable and generalizable.

By combining the IPD of relevant RCTs together and performing statistical analyses on the combined, patient-level data, we strive to raise the level of evidence regarding mesh prophylaxis for IH prevention after open AAA repair and to help identify those who will benefit most from this procedure. This can only be achieved through international collaboration. Despite the growing recognition of the ethical and scientific importance of data sharing and scientific transparency, one of the biggest challenges in undertaking IPD analyses relates to overcoming data-

For peer review only

sharing hurdles. Barriers range from successfully reaching original study authors; willingness or ability of authors to share data; and international ethics and regulations issues. For this study, collaboration will be initiated through the European Hernia Society (EHS). The EHS is an internationally recognized organization in the field of hernia surgery, and it has appointed a steering committee to oversee this IPDMA.

Current knowledge/data

Study	Types of surgery	Risk-ratio incidence of IH	Risk-ratio of reoperation for IH	Risk-ratio post- operative seroma	Risk ratio post-operative SSI
Indrakusuma et al. (2018) [17]	AAA open repair surgery	0.27 (0.11-0.66)	0.23 (0.05-1.05)	x	x
Aiolfi et al. (2022) [20]	All midline incisions	0.38 (0.24-0.58)	x	2.05 (1.35-3.13)	1.17 (0.82-1.67)
Jairam et al. (2020) [3]	All elective midline incisions	0.35 (0.21-0.57)	x	Onlay 2,23 (1,10 - 4,52)<br>Retromuscular 1,67 (0,81 – 3,47)	Onlay 1,67 (0,81 – 3,47)<br>Retromuscular 0,28 (0,10 – 0,82)

Table 1: Most recently published summary data of incisional hernia prevention by prophylactic mesh placement.

Aims

We aim to conduct a systematic review and IPDMA of RCTs, to evaluate the effectiveness of the use of a prophylactic mesh after open AAA surgery as compared to primary sutured closure with IH rate during long-term follow-up (2, 3, and 5-year IH rate) as primary outcome. Our secondary aims are to evaluate differences in postoperative complications within 30 days such as surgical site infection (SSI), surgical site occurrence (SSO) and fascial dehiscence, as well as pain (e.g., visual analogue scale (VAS) pain score, numeric rating scale (NRS) pain score), quality of life (e.g., EQ-4D, SF-36), and the need for re-operation (abdominal-wall and other) at different time points during follow-up (e.g., <30 days, 6 months, 1 year). Furthermore, we aim to identify potential subgroups of patients who will benefit most from prophylactic mesh reinforcement after open AAA surgery regarding the reduction in IH rate. The results of this study is assumed to support recommendations in future guideline updates, and they will directly inform clinicians regarding type of abdominal wall closure after open AAA repair. This will translate into benefit for those who will undergo AAA repair. Ultimately, reducing the incidence of IH after AAA repair is a socially responsible goal, as it will also result in reduced societal healthcare costs.

Methods and analysis

The basic study protocol was approved by the EHS scientific committee. Subsequently, it was submitted for registration to the International Prospective Register of Systematic Reviews (PROSPERO) (registration number CRD42022347881). It formed the basis for the present, detailed protocol, which was written in accordance with the Preferred Reporting Items for Systematic Reviews and Meta-Analyses Protocols (PRISMA) statement and PRISMA IPD (PRISMA-IPD) guidelines. Data transfer methods, developed in collaboration with the Erasmus MC data transfer office (DTO) and approved by the EHS, will guide the secure transfer and responsible use of IPD, adhering to current European data-sharing regulations.

Study identification

A literature search will be performed in the following databases: Cochrane Central Register of Controlled Trials (CENTRAL); MEDLINE Ovid (1946 onwards); Embase (1980 onwards); Web of Science Core Collection (1975

For peer review only

onwards) and Google Scholar. The search strategy will be tailor-made, by the investigators, together with an experienced, professional librarian from the Erasmus MC Medical Library. The complete search terms are noted in the supplementary files (Suppl. A).

Data procurement

For all identified studies, we will contact the corresponding author by email. If a current email address cannot be found or the author does not respond (up to three attempts), we will attempt to reach them by other means (phone, post, contact institution or any other means of contact that are available). Where IPD are available and authors or institutions are willing to share data, a data delivery agreement (DDA) will be drafted by both parties. A template DDA has been prepared for this study and it will be reasonably adapted if authors see the need to make changes to it, after which it will be signed. Dutch ethics regulations do not require explicit ethical approval for conducting IPDMAs. However, where local ethics regulations require it, ethics approval will be sought prior to sharing data. Pseudonymised or anonymised data sets (all formats will be acceptable, e.g., SPSS, Excel) and related data dictionaries will then be transferred and stored securely in a database at Erasmus MC, for use only as agreed on in the DDA. One original study investigator (first or senior author, at the discretion of the data owner) will be invited to be a co-author of the project if they are willing to assume responsibilities that meet authorship guidelines, as also stated in the DDA.

Data processing and validation

We will convert all data sets to a common format, combine data sets with a new variable identifying original trial and harmonize variables. Data checking will include evaluating baseline characteristics and results of comparisons for our main outcomes against results reported in original publications. We will also check for balancing of baseline participant characteristics in each treatment arm, and evaluate the extent to which all randomized participants in the IPD datasets have been included in study analyses. Authors will be consulted in the case of any inconsistencies or discrepancies. In cases where discrepancies cannot be resolved, we will (on a case-by-case basis) either conduct a sensitivity analysis with that study removed, or we will exclude the study from our analysis altogether.

Two independent investigators will parse data from all included published studies. From each study, we will extract the following data: country of study; funding source; study design; sample size; target population; inclusion/exclusion criteria; participant characteristics (age, sex, BMI, history of injury or surgery, comorbidities, medication use); type and context of intervention (e.g., mesh placement technique, type of mesh, imaging techniques used for the diagnosis of an IH, suture technique); AAA characteristics; pain and quality of life pre–post as available. For all patient-reported outcomes, we will extract the recall period in addition to the outcome. Where IPD are available, we will conduct all analyses using IPD instead of aggregate data, following data consistency checks described above.

Study quality assessment

Two investigators will independently evaluate risk of bias (ROB) for each included study using the Cochrane ROB tool, and disagreements will be resolved by a third investigator. Any authors involved in any included trial will not extract data from or assess the risk of bias in those trials. Duplicate publications will be identified to evaluate the trials and all available data simultaneously to maximize data extraction and correct bias assessment. The Cochrane ROB considers five domains of possible bias: randomization; deviations from intended interventions;

For peer review only

missing outcome data; measurement of the outcome and selection of the reported results. For each domain, ROB is rated as low, some concerns or high. The overall study will be considered to be of low ROB if all five domains are rated as low ROB, and high overall ROB if at least one domain is rated as high ROB or if some concerns are identified in multiple domains. We will consult authors of the original publications in the event of inadequate reporting or inconsistencies. If indicated, we will email the authors to request data that may not have been sufficiently included in the primary publication.

The following trial-related data will be extracted:

- Trial characteristics: bias risk components, trial design, period and number of sites, countries where the trial was conducted, number of intervention arms, length of follow-up and inclusion and exclusion criteria.
- Participant characteristics and comorbidities: number of randomized participants, analysed participants, participants lost to follow-up, mean age, age range, sex ratio, specific patient-based inclusion criteria, treatment characteristics (e.g., operating time).

Assessment of heterogeneity

Forest plots will be constructed to visualise and assess any signs of heterogeneity. Statistical heterogeneity will be assessed using the Chi-square test (threshold $p < .10$), the quantities of heterogeneity will be measured with the I^2 statistic, and possible heterogeneity will be assessed with relevant subgroup analyses.

All eligible patients from included RCTs will be included for final analysis if meeting the following criteria: adults (18 years or older), diagnosed with AAA using any common method (e.g., radiographs, CT, clinical criteria, diagnosis by a healthcare professional). Additionally, inclusion criteria from included RCTs will be evaluated and the criteria of the IPDMA will be amended if required. A potential subject who meets any of the following criteria will be excluded from final analysis: emergency surgery or the presence of a mesh in the abdominal wall on the midline from previous hernia repair. Additionally, exclusion criteria from included RCTs will be evaluated and the criteria of the IPDMA will be amended accordingly.

Sample size calculations stated in the included studies will be assessed. New power calculations will be performed for subgroup analyses that are performed on IPD. A one-stage meta-analysis of IPD will be performed on the data received from the different included studies, which were identified through the literature search. We will conduct time-to-event analysis for all included patients using Cox regression analysis with trial and centre (nested under trial) as cluster terms to compare groups with and without the placement of the prophylactic mesh by the use of the hazard ratio and the corresponding two-sided 95% confidence interval [21]. Risk factors will be evaluated using Cox-regression analysis. Comparison of categorical and continuous variables between groups will be performed using mixed logistic regression analysis with, but not limited to, baseline value, age, gender, and operation indication as possible covariates and trial and centre (nested under trial) as random effects.

Missing data

To avoid bias induced by ignoring missing data in clinical research, it is widely acknowledged that imputation techniques can be considered to replace missing values. We anticipate that the proportion of missing values for the primary and secondary outcomes will be less than 5%, in trials that documented these parameters, and therefore we will consider imputation. For partially missing data, traditional multiple imputation techniques will be

For peer review only

performed per individual dataset, if not yet done by the researchers from the study. But also, if the proportion of missing values in relation to the total dataset is reasonably small allowing for the construction of a robust imputation model. However, in a secondary analysis, we will consider using multiple imputation and/or best-worst and worst-best case scenarios if we can't ignore missing data. We will describe the proportion of missing values for each dataset included in the IPDMA.

Treatment efficacy

To evaluate treatment efficacy, we want to employ a one-step meta-analysis on the primary outcome parameter, which is IH rate. This will be evaluated using a time-to-event analysis. All data will be harmonized in one large dataset and analysed as pooled outcome data of all included patients in the different RCTs, controlling for stratification per centre (indicated by an additional unique covariate for each of the different trials). We will analyse the effect of the treatment by intention to treat, regardless of the methods used in the original study. Cox regression analysis stratified per trial (on randomisation level) will be used to assess mesh efficacy for preventing IH occurrence. Effect sizes will be documented with relative risk (RR, 95% CI). For the secondary outcome measure postoperative complications within 30 days, such as SSI, SSO, fascial dehiscence, and the need for re-operation (abdominal and other) at different time points during follow-up (e.g., <30 days, 6 months, 1 year), we will conduct logistic regression models accounting for clustering on trial level. Effect sizes will be documented with odds ratios (OR, 95%CI). For the secondary outcome measure pain and quality of life we will use linear regression models accounting for clustering on the trial level as well and effect sizes will be documented with regression coefficients (β, 95%CI).

If a one-stage meta-analysis is not feasible, we will conduct a two-stage meta-analysis where we will first analyse each trial separately, and then pool results across trials. In step 1, within each trial, we will evaluate the effect of assigned intervention by intention to treat, regardless of method used in the original study. If study heterogeneity prevents us from harmonising data, then we will navigate this using a statistical approach based on available data. This will likely involve transforming data into standardised means differences or applying proportion of maximum scaling methods.

In studies where we are unable to obtain IPD, we will extract aggregate data from published manuscripts as they are reported in the published articles. Similar models will be performed for secondary outcomes as data permit. In cases of dichotomous outcomes, we will perform binary modelling and report effect sizes as relative risk (RR, 95% CI).

In step 2, we will perform random effects meta-analysis employing restricted maximum likelihood. We will report study heterogeneity as I^2 and τ^2. In cases of notable heterogeneity (I^2 >50%), we will consider possible sources such as study design, treatment duration, comparison treatment, treatment adherence or study quality. We will then consider performing meta-regression, subgroup analysis or sensitivity analyses to explain or account for these potential sources of heterogeneity. We will pool results of studies both with and without IPD data after verifying that effect sizes of IPD studies do not differ from non-IPD studies.

Hypotheses

For the primary research question, it is hypothesized that prophylactic mesh reinforcement reduced IH rate in comparison to primary sutured closure. Our secondary hypotheses are that postoperative complications such as

For peer review only

SSI, and SSO rate are comparable for the two methods of abdominal closure, while we hypothesize that prophylactic mesh reinforcement is superior regarding fascial dehiscence, pain, quality of life and the need for re-operation (abdominal-wall and other) as compared with the primary suture group.

Treatment effect-modifier analyses

We will conduct treatment effect-modifier analyses to identify subgroups of individuals undergoing open-AAA surgery who benefit most from the placement of a prophylactic mesh by including interaction terms between subgroup and treatment group in the corresponding regression analyses. We have proposed several subgroup characteristics that we hypothesize may modify the effect of the prophylactic mesh on our main outcome (IH formation), based on expert opinion. These proposed subgroups include the following baseline characteristics: (1) BMI score (Patients with a higher BMI are at a higher risk for the development of an IH); (2) Primary fascial closure with different SL/WL ratio's (A higher suture length (SL) to wound length (WL) ratio results in less IH's and therefore the use of different SL/WL ratio's might result in wrong conclusions and/or recommendations); (3) Patients with connective tissue disorders (can be associated with the formation of the AAA and also the healing of the abdominal wall and therefore the formation of an IH).

Patient and Public Involvement

No patient involvement was sought for the development of the protocol for this IPDMA.

Ethics and dissemination

No new data will be collected in this study. We will adhere to institutional, national and international regulations regarding the secure and confidential sharing of IPD, addressing ethics as indicated. We intend to publish the IPDMA in a peer reviewed journal.

Handling and storage of data and documents

Patient data from the participating centres where the RCTs were held, will be anonymized, and transferred via encrypted and secure data transfer. Before data transfer, a data delivery agreement will be signed by both parties. The EHS will handle and store data as an independent party. Only the assigned researcher in the Erasmus MC will have access to the data. No sponsor is present for the study.

Acknowledgments

We thank dr. W.M. Bramer at the ErasmusMC University Medical Centre Medical Library for assisting us with developing our search terms and managing our search.

Author contributions

All authors were involved in the study design and all will contribute to the interpretation of the results. RB contacted the potential data deliverers, will coordinate the data collection, and perform/ supervise the data analyses. RB wrote the manuscript together with FD and PT. RB and FD will have full access to the study data. RB, FD, CB, CS, MM, PB, JE, EB, SH, FB, OD, DS, FM, PT and the European Hernia Society Prophylactic Mesh Study Group Collaborators approved the final manuscript.

Word count: 2977

For peer review only

References

1. Azin A, Hirpara D, Jackson T, Okrainec A, Elnahas A, Chadi SA, Quereshy FA. Emergency laparoscopic and open repair of incarcerated ventral hernias: a multi-institutional comparative analysis with coarsened exact matching. Surg Endosc. 2019 Sep;33(9):2812-2820.

2. Dadashzadeh ER, Huckaby LV, Handzel R, Hossain MS, Sanin GD, Anto VP, Bou-Samra P, Moses JB, Cai S, Phelos HM, Simmons RL, Rosengart MR, van der Windt DJ. The Risk of Incarceration During Nonoperative Management of Incisional Hernias: A Population-based Analysis of 30,998 Patients. Ann Surg. 2022 Feb 1;275(2):e488-e495.

3. Jairam AP, Lopez-Cano M, Garcia-Alamino JM, Pereira JA, Timmermans L, Jeekel J, et al. Prevention of incisional hernia after midline laparotomy with prophylactic mesh reinforcement: a meta-analysis and trial sequential analysis. BJS Open. 2020;4(3):357-68.

4. van Ramshorst GH, Eker HH, Hop WC, Jeekel J, Lange JF. Impact of incisional hernia on health-related quality of life and body image: a prospective cohort study. Am J Surg. 2012;204(2):144-50.

5. van Ramshorst GH, Eker HH, van der Voet JA, Jeekel J, Lange JF. Long-term outcome study in patients with abdominal wound dehiscence: a comparative study on quality of life, body image, and incisional hernia. J Gastrointest Surg. 2013;17(8):1477-84.

6. Gillion JF, Sanders D, Miserez M, Muysoms F. The economic burden of incisional ventral hernia repair: a multicentric cost analysis. Hernia. 2016;20(6):819-30.

7. Carney MJ, Weissler JM, Fox JP, Tecce MG, Hsu JY, Fischer JP. Trends in open abdominal surgery in the United States-Observations from 9,950,759 discharges using the 2009-2013 National Inpatient Sample (NIS) datasets. Am J Surg. 2017;214(2):287-92.

8. Rahbari NN, Knebel P, Diener MK, Seidlmayer C, Ridwelski K, Stoltzing H, et al. Current practice of abdominal wall closure in elective surgery - Is there any consensus? BMC Surg. 2009;9:8.

9. Valeriy Shubinets JPF, Michael A Lanni, Michael G Tecce, Eric M Pauli, William W Hope, Stephen J Kovach, John P Fischer. Incisional Hernia in the United States: Trends in Hospital Encounters and Corresponding Healthcare Charges. Am Surg. 2018;1:118-25.

10. Abo-Ryia MH, El-Khadrawy OH, Abd-Allah HS. Prophylactic preperitoneal mesh placement in open bariatric surgery: a guard against incisional hernia development. Obes Surg. 2013;23(10):1571-4.

11. Gutierrez de la Pena C, Medina Achirica C, Dominguez-Adame E, Medina Diez J. Primary closure of laparotomies with high risk of incisional hernia using prosthetic material: analysis of usefulness. Hernia. 2003;7(3):134-6.

12. Pans A, Elen P, Dewe W, Desaive C. Long-term results of polyglactin mesh for the prevention of incisional hernias in obese patients. World J Surg. 1998;22(5):479-82; discussion 82-3.

13. Strzelczyk JM, Szymanski D, Nowicki ME, Wilczynski W, Gaszynski T, Czupryniak L. Randomized clinical trial of postoperative hernia prophylaxis in open bariatric surgery. Br J Surg. 2006;93(11):1347-50.

14. Wanhainen A, Verzini F, Van Herzeele I, Allaire E, Bown M, Cohnert T, et al. Editor's Choice - European Society for Vascular Surgery (ESVS) 2019 Clinical Practice Guidelines on the Management of Abdominal Aorto-iliac Artery Aneurysms. Eur J Vasc Endovasc Surg. 2019;57(1):8-93.

For peer review only

15. Jairam AP, Timmermans L, Eker HH, Pierik R, van Klaveren D, Steyerberg EW, et al. Prevention of incisional hernia with prophylactic onlay and sublay mesh reinforcement versus primary suture only in midline laparotomies (PRIMA): 2-year follow-up of a multicentre, double-blind, randomised controlled trial. Lancet. 2017;390(10094):567-76.

16. Deerenberg EB, Henriksen NA, Antoniou GA, Antoniou SA, Bramer WM, Fischer JP, et al. Updated guideline for closure of abdominal wall incisions from the European and American Hernia Societies. Br J Surg. 2022.

17. Indrakusuma R, Jalalzadeh H, van der Meij JE, Balm R, Koelemay MJW. Prophylactic Mesh Reinforcement versus Sutured Closure to Prevent Incisional Hernias after Open Abdominal Aortic Aneurysm Repair via Midline Laparotomy: A Systematic Review and Meta-Analysis. Eur J Vasc Endovasc Surg. 2018;56(1):120-8.

18. Dewulf M, Muysoms F, Vierendeels T, Huyghe M, Miserez M, Ruppert M, et al. Prevention of Incisional Hernias by Prophylactic Mesh-augmented Reinforcement of Midline Laparotomies for Abdominal Aortic Aneurysm Treatment: Five-year Follow-up of a Randomized Controlled Trial. Ann Surg. 2022;276(4):e217-e22.

19. Jairam AP, Timmermans L, Eker HH, Pierik RE, van Klaveren D, Steyerberg EW, et al. Long Term Follow-up PRIMA Trial. [Updated 2022 Jul 18]. Available from: https://onderzoekmetmensen.nl/nl/trial/22792.

20. Aiolfi, A., et al. Prophylactic mesh reinforcement for midline incisional hernia prevention: systematic review and updated meta-analysis of randomized controlled trials. Hernia. 2023 May; 27(2): 213-224.

21. de Jong VMT, Moons KGM, Riley RD, Tudur Smith C, Marson AG, Eijkemans MJC, Debray TPA. Individual participant data meta-analysis of intervention studies with time-to-event outcomes: A review of the methodology and an applied example. Res Synth Methods. 2020 Mar;11(2):148-168.

For peer review only

Suppl. A: Search strategies of the used databases

medline ALL Ovid

(Aortic Aneurysm, Abdominal / OR Aortic Aneurysm / OR ((Aneurysm /) AND Aorta, Abdominal /) OR ((aort* ADJ3 aneurysm*) OR aaa).ab,ti.) AND (Surgical Mesh / OR (mesh* OR dynamesh* OR vitamesh* OR surgimesh*).ab,ti.) AND (exp Preventive Health Services / OR prevention.fx. OR (prevent* OR prophyla* OR augment* OR reinforce*).ab,ti.)

embase.com

('abdominal aortic aneurysm'/exp OR 'aortic aneurysm'/de OR ((aneurysm/de OR 'aneurysm surgery'/de) AND 'abdominal aorta'/de) OR ((aort* NEAR/3 aneurysm*) OR aaa):Ab,ti) AND ('surgical mesh'/exp OR (mesh* OR dynamesh* OR vitamesh* OR surgimesh*):ab,ti) AND (prophylaxis/de OR prevention/de OR prevention:lnk OR (prevent* OR prophyla* OR augment* OR reinforce*):ab,ti)

Web of science

TS=((((aort* NEAR/2 aneurysm*) OR aaa)) AND ((mesh* OR dynamesh* OR vitamesh* OR surgimesh*)) AND ((prevent* OR prophyla* OR augment* OR reinforce*)))

Cochrane CENTRAL

(((aort* NEAR/3 aneurysm*) OR aaa):Ab,ti) AND ((mesh* OR dynamesh* OR vitamesh* OR surgimesh*):ab,ti) AND ((prevent* OR prophyla* OR augment* OR reinforce*):ab,ti)

Google scholar

"aortic|aorta aneurysm|aneurysms" mesh|dynamesh|vitamesh|surgimesh preventive|prevention|prophylaxis|prophylactic|augmentation|reinforcement

For peer review only

This checklist originates from Table 3 in Moher D et al: Preferred reporting items for systematic review and meta-analysis protocols (PRISMA-P) 2015 statement. Systematic Reviews 2015 4:1.

Section/topic	#	Checklist item	Information reported Yes	Information reported No	Page number(s)
**ADMINISTRATIVE INFORMATION**					
**Title**					
Identification	1a	Identify the report as a protocol of a systematic review	X		1
Update	1b	If the protocol is for an update of a previous systematic review, identify as such		X	
**Registration**	2	If registered, provide the name of the registry (e.g., PROSPERO) and registration number in the Abstract	X		2
**Authors**					
Contact	3a	Provide name, institutional affiliation, and e-mail address of all protocol authors; provide physical mailing address of corresponding author	X		1
Contributions	3b	Describe contributions of protocol authors and identify the guarantor of the review	X		1
**Amendments**	4	If the protocol represents an amendment of a previously completed or published protocol, identify as such and list changes; otherwise, state plan for documenting important protocol amendments		X	
**Support**					
Sources	5a	Indicate sources of financial or other support for the review	X		1
Sponsor	5b	Provide name for the review funder and/or sponsor	X		1
Role of sponsor/funder	5c	Describe roles of funder(s), sponsor(s), and/or institution(s), if any, in developing the protocol	X		1
**INTRODUCTION**					
**Rationale**	6	Describe the rationale for the review in the context of what is already known	X		3
**Objectives**	7	Provide an explicit statement of the question(s) the review will address with reference to participants, interventions, comparators, and outcomes (PICO)	X		3
**METHODS**					
**Eligibility criteria**	8	Specify the study characteristics (e.g., PICO, study design, setting, time frame) and report characteristics (e.g., years considered, language, publication status) to be used as criteria for eligibility for the review	X		4
**Information sources**	9	Describe all intended information sources (e.g., electronic databases, contact with study authors, trial registers, or other grey literature sources) with planned dates of coverage	X		4
**Search strategy**	10	Present draft of search strategy to be used for at least one electronic database, including planned limits, such that it could be repeated	X		12
STUDY RECORDS					
Data management	11a	Describe the mechanism(s) that will be used to manage records and data throughout the review	X		8
Selection process	11b	State the process that will be used for selecting studies (e.g., two independent reviewers) through each phase of the review (i.e., screening, eligibility, and inclusion in meta-analysis)	X		5
Data collection process	11c	Describe planned method of extracting data from reports (e.g., piloting forms, done independently, in duplicate), any processes for obtaining and confirming data from investigators	X		5
**Data items**	12	List and define all variables for which data will be sought (e.g., PICO items, funding sources), any pre-planned data assumptions and simplifications	X		5
**Outcomes and prioritization**	13	List and define all outcomes for which data will be sought, including prioritization of main and additional outcomes, with rationale	X		5
**Risk of bias in individual studies**	14	Describe anticipated methods for assessing risk of bias of individual studies, including whether this will be done at the outcome or study level, or both; state how this information will be used in data synthesis	X		5
DATA					
**Synthesis**	15a	Describe criteria under which study data will be quantitatively synthesized	X		5
	15b	If data are appropriate for quantitative synthesis, describe planned summary measures, methods of handling data, and methods of combining data from studies, including any planned exploration of consistency (e.g., I^2, Kendall's tau)	X		6
	15c	Describe any proposed additional analyses (e.g., sensitivity or subgroup analyses, meta-regression)	X		7-8
	15d	If quantitative synthesis is not appropriate, describe the type of summary planned		X	
**Meta-bias(es)**	16	Specify any planned assessment of meta-bias(es) (e.g., publication bias across studies, selective reporting within studies)		X	
**Confidence in cumulative evidence**	17	Describe how the strength of the body of evidence will be assessed (e.g., GRADE)		X	
